# A New Form of Suture

**Published:** 1867-08

**Authors:** D. Prince

**Affiliations:** Jacksonville, Ill.


### A NEW FORM OF SUTURE.

Mr. Editor:—My attention has just been attracted by an article under the above heading, in the April No. of the Journal, p. 150.

The modification of the twisted suture, in which a ring of rubber is substituted for thread or or other material, is figured and described by Dr. Washington L. Atlee, in the *American Journal of the Medical Sciences* for January, 1860, page 81. He claimed it as *original*, and thought that it would *displace all other sutures.*

The fault of the suture is, that if it pulls enough it pulls too much, and if it does not pull too much it does not pull enough.

This will be plain enough, with a little explanation. The theory on which rubber is employed in preference to thread or other inelastic material is, that elastic rubber will yield on the occurrence of swelling, thus obviating the severe pressure to which the included parts must be subjected when inelastic materials are employed.

It must be obvious, on a little reflection, that if the rubber is strong enough to hold the parts in contact, it will pull all the time without any swelling, and this is pulling too much. And, on the other hand, if it does not pull enough to subject the parts to an unnecessary pressure without swelling, it does not pull enough to hold the surfaces in contact. The reason of this is, that rubber, when applied tense, tends to shorten itself, while inelastic material holds the parts in contact without tending to shorten itself. It, therefore, does not press at all within the distance of its original application.

Again, the rubber bridge over the wound will not slide out at its abutments upon the pin. All the yielding that can be expected of it is in a direction perpendicular to the surface, and here, if the material is so weak as to do this readily, it is an insecure fastening; and if it is strong enough to be secure, it is too strong, for it must press unnecessarily without any swelling at all.

The employment of this suture in varicose veins, is an improvement over the ordinary twisted suture, because the fastening still draws, after the parts yield somewhat. This quality is not desired in effecting union of divided surfaces.

It is probable that the best modification of the twisted suture is, to employ a strip of plaster. When convenient, the plaster can be put on first and the pin pushed through from side to side, and if the tissues are hard, the introduction of an exploring needle, as a guide, will aid the process very much. In many cases, the pin must be pushed through first. Introduce the pin through the plaster and then along the groove of the exploring needle, and while the point of the needle is on a level with the surface of the skin, draw the plaster firmly over across the wound and push the point of the needle through it. This can be fortified by a thread, in the manner of an ordinary twisted suture, but the additional security will rarely be necessary. This presents such a broad surface that constriction and ulceration are not likely to occur. If the parts swell, the pin must be rotated and withdrawn, which can be done without disturbing the plaster. Yours truly,

*Jacksonville, June 26, 1867.* D. PRINCE.

